# Effects of a social stimulus on gene expression in a mouse model of fragile X syndrome

**DOI:** 10.1186/s13229-017-0148-6

**Published:** 2017-06-23

**Authors:** Tiffany D. Rogers, Allison M. J. Anacker, Travis M. Kerr, C. Gunnar Forsberg, Jing Wang, Bing Zhang, Jeremy Veenstra-VanderWeele

**Affiliations:** 1 Department of Psychiatry, Vanderbilt University, 7158 MRBIII, 465 21st Avenue South, Nashville, TN 37232 USA; 2 Department of Psychology, Middle Tennessee State University, 355 Jones Hall, 624 Old Main Circle, Murfreesboro, TN 37132 USA; 3 Department of Psychiatry, Columbia University; New York State Psychiatric Institute, 1051 Riverside Dr, Unit 78, New York, NY 10032 USA; 4 The University of Tennessee Health Science Center College of Medicine, 910 Madison Ave, Suite 1002, Memphis, TN 38163 USA; 5 College of Medicine, Medical University of South Carolina, Charleston, SC 29425 USA; 6 Lester and Sue Smith Breast Center, Baylor College of Medicine, Houston, TX 77030 USA; 7 Department of Molecular and Human Genetics, Baylor College of Medicine, Houston, TX 77030 USA

**Keywords:** Fragile X syndrome, Autism spectrum disorder, Social behavior, RNA sequencing, Amygdala, Prefrontal cortex

## Abstract

**Background:**

People with fragile X syndrome (FXS) often have deficits in social behavior, and a substantial portion meet criteria for autism spectrum disorder. Though the genetic cause of FXS is known to be due to the silencing of *FMR1*, and the *Fmr1* null mouse model representing this lesion has been extensively studied, the contributions of this gene and its protein product, FMRP, to social behavior are not well understood.

**Methods:**

*Fmr1* null mice and wildtype littermates were exposed to a social or non-social stimulus. In one experiment, subjects were assessed for expression of the inducible transcription factor c-Fos in response to the stimulus, to detect brain regions with social-specific activity. In a separate experiment, tissue was taken from those brain regions showing differential activity, and RNA sequencing was performed.

**Results:**

Immunohistochemistry revealed a significantly greater number of c-Fos-positive cells in the lateral amygdala and medial amygdala in the brains of mice exposed to a social stimulus, compared to a non-social stimulus. In the prelimbic cortex, there was no significant effect of social stimulus; although the number of c-Fos-positive cells was lower in the social condition compared to the non-social condition, and negatively correlated with c-Fos in the amygdala. RNA sequencing revealed differentially expressed genes enriched for molecules known to interact with FMRP and also for autism-related genes identified in the Simons Foundation Autism Research Initiative gene database. Ingenuity Pathway Analysis detected enrichment of differentially expressed genes in networks and pathways related to neuronal development, intracellular signaling, and inflammatory response.

**Conclusions:**

Using the *Fmr1* null mouse model of fragile X syndrome, we have identified brain regions, gene networks, and molecular pathways responsive to a social stimulus. These findings, and future experiments following up on the role of specific gene networks, may shed light on the neural mechanisms underlying dysregulated social behaviors in fragile X syndrome and more broadly.

**Electronic supplementary material:**

The online version of this article (doi:10.1186/s13229-017-0148-6) contains supplementary material, which is available to authorized users.

## Background

Social impairment is one of the two core symptom domains in autism spectrum disorder (ASD) [1], but we have limited understanding of the mechanisms underlying this impairment. One way to probe the molecular underpinnings of social difficulties in ASD is to study single gene syndromes that frequently include prominent ASD symptoms, such as fragile X syndrome (FXS), which is found in about 1% of children with ASD [2–4]. Between one quarter and two thirds of children with FXS meet criteria for ASD, depending upon the study [5–15]. In some but not all studies, the average child with FXS and autism spectrum disorder (FXS-ASD) has fewer ritualistic behaviors and perhaps fewer social deficits than the average child with autism spectrum disorder without FXS [10, 16–20], but these differences are not large enough to identify a behaviorally defined subgroup without genetic testing.

FXS is caused by expansion of a trinucleotide repeat in the 5′ untranslated region of the *FMR1* gene leading to gene hypermethylation and transcriptional silencing. *Fmr1* null mice have been a primary tool to investigate the cellular, synaptic, and molecular changes underlying FXS [21]. These mice display behavioral, structural, and molecular abnormalities that mirror some of those seen in FXS [22]. While the preponderance of evidence suggests that *Fmr1* null mice do have changes in social behavior compared to wildtype controls, results have been inconsistent across labs and appear to be dependent on the inbred strain background of the mice being studied, with abnormalities more frequently reported on an FVB/129S mixed background [22–25]. The most common finding is a loss of preference for a novel mouse over a familiar mouse, but some laboratories have also reported that *Fmr1* mice fail to prefer a novel mouse over a novel object, show diminished free social interactions, or are more likely to back out of the tube test for dominance [23–43]. In addition to abnormal social behavior, *Fmr1* null mice have been reported to show several other behavioral deficits, including hyperactivity, attention deficits, learning deficits, and abnormal reactions to sensory stimuli [25, 44–47], although these behavioral changes have also been inconsistent across labs.

Many studies have focused on the molecular consequences of *FMR1* loss with the goal of developing an effective treatment for FXS. Considerable data support a critical role for the gene’s protein product, FMRP, downstream of group 1 metabotropic glutamate receptor (mGluR) signaling, leading to the mGluR theory of fragile X [48]. Although improvement of behavioral and synaptic abnormalities was observed in animal models following treatment with mGluR5 negative allosteric modulators [49, 50], clinical trials have not yet yielded benefit in adolescents or adults with FXS [51]. Other potential treatments have also been studied, including lithium [52], minocycline [53], and arbaclofen [54], but no treatment has yet shown consistent improvement in behavior in the human population.

Despite extensive characterization of the *Fmr1* null mouse, the role of FMRP in mediating social behavior remains relatively unknown. The aim of the current investigation was to use the *Fmr1* (FVB/129) null mouse as a tool to further investigate molecular changes that may impact social behavior. We designed a series of experiments to understand the brain areas involved in response to social stimuli in these animals and to identify changes in levels of RNA expression that may identify molecular pathways altered in this model. Beyond insight into potential mechanisms underlying altered social behavior in the *Fmr1* null mouse, we hoped to use this well-studied model as a proof-of-concept of this use of social stimuli to identify genes and pathways that can be cross-validated with other data from FXS and ASD.

## Methods

### Animals

Male *Fmr1* null mice [55] (*Fmr1* null, FVB.129P2-Pde6b + Tyrc-ch Fmr1tm1Cgr/J; stock #4624) and wildtype mice (WT, FVB.129P2-Pde6b + Tyrc-ch/AntJ; stock #4828) were purchased from Jackson Laboratories. These wildtype mice were generated from the *Fmr1* line and are considered the standard control for *Fmr1* null mice, as have been used in behavioral and molecular studies [56, 57]. Mice were housed two to five to a cage in the Vanderbilt Neurobehavioral core with a 12-h light/dark cycle and food and water available ad libitum. All mice were between 8 and 14 weeks of age for all experiments. Behavioral experiments commenced following a habituation period of at least 1 week at the Vanderbilt Neurobehavioral core.

### Home cage social exposure for immunohistochemistry

We first tested the classic 3-chamber sociability apparatus, as implemented by the Crawley lab [58], for social exposures in wildtype C57BL/6J mice. During these experiments, the social exposure was conducted in a single chamber containing a stimulus mouse in an inverted pencil cup. The non-social exposure was conducted in a single chamber containing only an inverted pencil cup. Using this approach, we found widespread c-Fos activation that did not easily separate between the social and non-social condition (data not shown), similar to a recent study performed in juvenile C57BL/6 mice [59] which found only one brain area (basolateral amygdala) within which c-Fos activation was differentiated between social and non-social groups. We therefore modified this task to optimize the detection of social-specific c-Fos activation in a separate set of C57BL/6J mice. First, each mouse was tested within a standard housing cage to prevent a ceiling effect of c-Fos activation from the novel, open 3-chamber apparatus. Second, in order to control for exposure to novelty, mice were habituated to all components of the stimulus (cage, pencil cup) except for the social stimulus, which was controlled for using a toy horse (Playmobil) as a novel, non-social stimulus. After initial experiments in C57BL/6J animals, *Fmr1* null and wildtype mice (*n* = 16, four per stimulus per genotype) were used in the behavioral experiments described here.

**Fig. 1:**
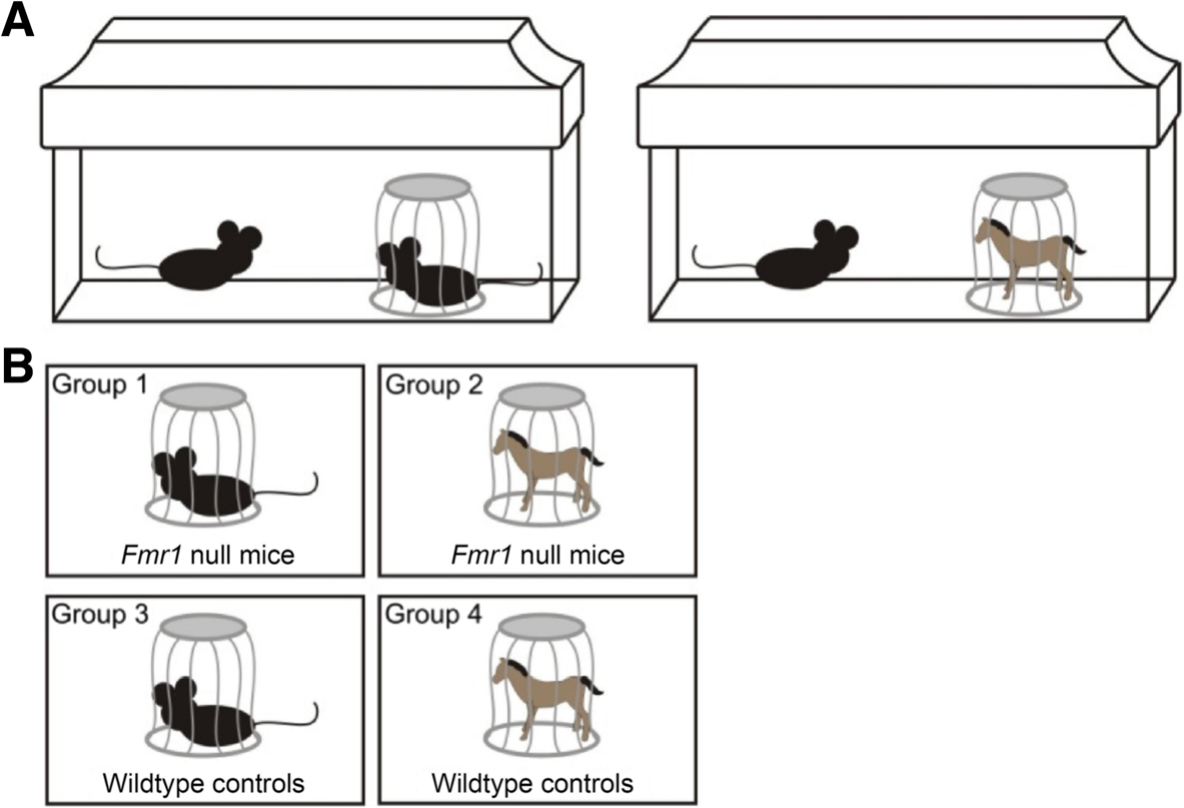
Experimental conditions and groups. **a** Mice were placed in a cage with an empty pencil cup 3 days prior to testing. At the time of the test, a social stimulus (*left*) or non-social stimulus (*right*) was placed in the pencil cup for 30 min. **b** There were four testing groups: Fmr1 KO with social or non-social stimulus exposure, or wildtype controls with social or non-social stimulus exposure

To habituate mice to the pencil cup stimulus, each mouse was individually separated into a novel cage that contained an empty pencil cup 3 days prior to the behavioral experiments (see Fig. 1a). After 3 days of habituation, each group of four mice (a “quad”), composed of two wildtype mice and two mutant mice, with each mouse separately housed, was placed in a room within the Vanderbilt Neurobehavioral core. The mice were allowed to habituate to the room for 1 h. A social stimulus, a novel mouse, was then placed in the pencil cups of one wildtype and one mutant cage, and a non-social stimulus, a toy horse (Playmobil), was placed in the pencil cups of the remaining wildtype and mutant cage (Fig. 1b). The exposure period lasted 30 min.

### Tissue harvest for immunohistochemistry

Tissue harvest was conducted 2 h after the conclusion of the behavioral exposure period to allow for expression of c-Fos. As previously described [60], each mouse was deeply anesthetized with pentobarbital (Nembutal, Lundbeck, Deerfield, IL) and then intracardially perfused with 4% paraformaldehyde (ph~7.4). Whole brains were harvested and stored in 4% paraformaldehyde for 24 h and then in 30% sucrose for at least 24 h. Each brain was sectioned on a sliding microtome into 40 μm sections. Sections were stored in a freezing solution (PBS, ethylene glycol, and glycerol) until staining.

### Immunohistochemistry

**Fig. 2:**
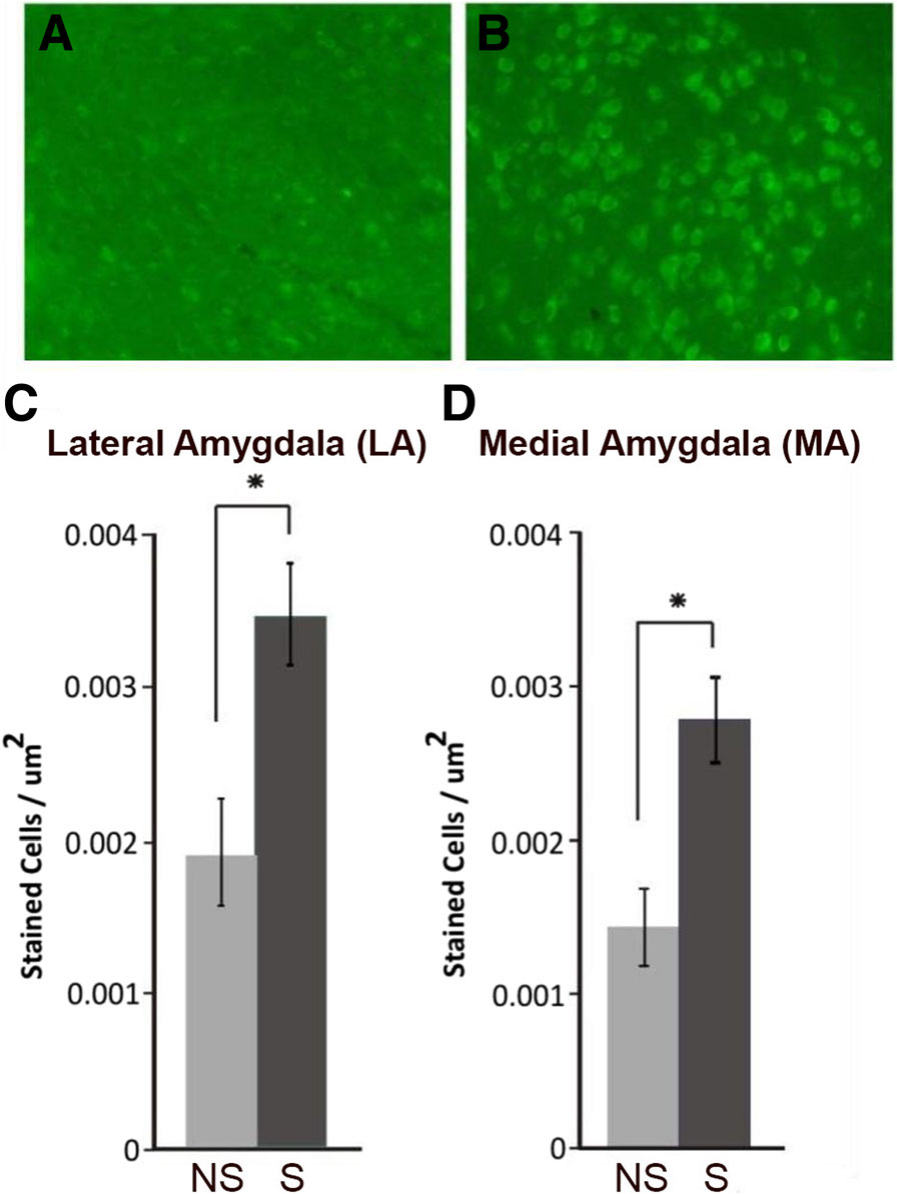
c-Fos staining in C57BL/6J mice. The number of cells was greater in the social (S) condition (**b**) compared to the non-social (NS) condition (**a**) in the lateral/basolateral amygdala (**c**), and the medial amygdala (**d**). *p < 0.05

Initial staining was performed in C57BL/6J mice following the behavioral and tissue harvest methods described above. Multiple brain areas including the orbital cortex, frontal association area, prelimbic cortex, cingulate cortex, insular cortex, accumbens core, accumbens shell, caudate putamen, peduncular cortex, thalamic nuclei, hypothalamic nuclei, medial amygdala, basolateral amygdala, CA1 of the hippocampus, CA2 of the hippocampus, CA3 of the hippocampus, central amygdaloid nucleus, oriens layer of hippocampus, lateral amygdaloid nucleus, zona incerta, parietal association area, subthalamic nucleus, enterohinal cortex, mammullary nuclei, and perihinal cortex were examined for differential expression of c-Fos by counting stained cells. In these mice, we found that the areas with the most significant differential c-Fos staining in the social versus novel object comparison were the medial amygdala and lateral amygdala (Fig. 2). These brain areas were then selected as target areas for staining and cell counting in the *Fmr1* null and wildtype control mice. Early in the cell counting procedure for *Fmr1* mice, we visually scanned regions listed above and observed what appeared to be differential expression of c-Fos within the prelimbic cortex (PLC) in wildtype control animals. Because of these expression differences and the functional relationship between the amygdala and prefrontal cortex, c-Fos expression within the PLC was included in *Fmr1* analyses.

Brain sections containing the medial and lateral amygdala and prefrontal cortex were stained for c-Fos expression in the *Fmr1* null and wildtype control animals with each genotype and stimulus group represented evenly in each batch of staining. As described previously [60], brain sections were washed in 0.1 M phosphate buffer (PB, pH~7.8) for 20 min, 0.05 M Tris-buffered saline (TBS, pH~7.6) for 20 min, and 0.05 M tris-buffered saline with 0.4% triton-x (TBST, pH~7.6) for 20 min. Sections were then placed in a c-Fos rabbit anti-mouse primary antibody (1:2000) in TBST for 60 h at 6 °C. Four washes in TBST for 15 min preceded a donkey anti-rabbit secondary antibody (1:200) in TBST for 2 h. Sections were then washed in TBST and TBS for 15 min each and washed twice in tris-buffer (TB, pH~8.2) for 15 min. Brain sections were then mounted on slides and covered. Using a Zeiss Axio Imager.M2 microscope and AxioVision 4.8 software, multiple 5× pictures of each brain section were acquired, stitched together, and converted to a single image per slice (Carl Zeiss, Inc., Thornwood, NY, USA). An anterior/posterior (AP) coordinate was assigned to each slice according to the Franklin and Paxinos mouse atlas [61]. Brain sections containing areas of interest were matched across the quad according to AP coordinate labels and 20× pictures were taken for cell counting.

### Cell counting

**Fig. 3:**
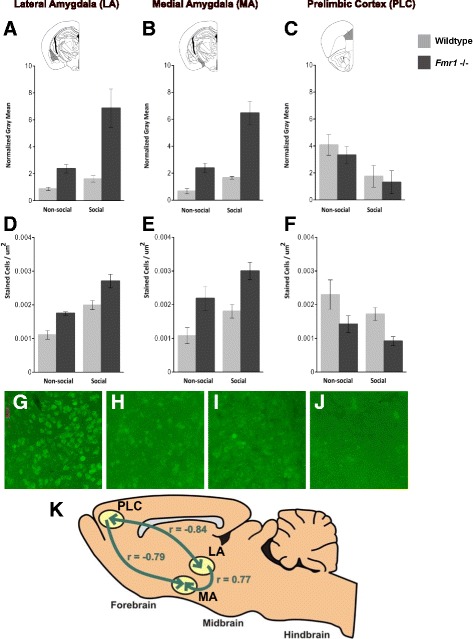
c-Fos staining in *Fmr1* mice. The intensity of c-Fos staining (**a**–**c**) and the number of c-Fos-positive cells (**d**–**f**) in the lateral amygdala (**a**, **d**), medial amygdala (**b**, **e**), and prelimbic cortex (**c**, **f**). *Insets* show anatomical regions assessed shaded in gray. Representative staining for c-Fos in the PLC of (**g**) wildtype mouse exposed to a non-social stimulus, (**h**) wildtype mouse exposed to a social stimulus, (**l**) *Fmr1* KO mouse exposed to a non-social stimulus, and (**j**) *Fmr1* KO mouse exposed to a social stimulus. The number of c-Fos-positive cells was compared between brain regions for each subject. There was a significant positive correlation between LA and MA, and a significant negative correlation between each amygdalar region and the PLC (**k**)

We used the Franklin and Paxinos mouse atlas [61] to select regions for cell counting. Following the methods of Hale and colleagues [62], we selected ten rostrocaudal levels ranging from −0.94 to −2.06 mm from bregma for counting c-Fos stained cells in the medial and lateral/basolateral amygdala. All medial amygdala (MA) cell counts were within the anatomical boundaries of the medial amygdala nucleus anterior dorsal, the medial amygdala nucleus anteroventral, the medial amygdala nucleus anterior, the medial amygdala nucleus posterodorsal, and the medial amygdala nucleus posteroventral. All lateral/basolateral amygdala (LA) cell counts were within the anatomical boundaries of the lateral amygdaloid nucleus, the basolateral amygdaloid nucleus anterior, the lateral amygdaloid nucleus dorsolateral, the lateral amygdaloid nucleus ventrolateral, the basolateral amygdaloid nucleus posterior, and the lateral amygdaloid nucleus ventromedial. For cell counts within the prefrontal cortex, we used 11 rostrocaudal levels ranging from 2.68 to 1.54 mm from bregma and used the anatomical borders of the prelimbic area (PLC; Fig. 3). In all areas, cells were counted from the left and right hemispheres and averaged. The area of each selected region was also measured with NIH ImageJ software [63] to determine number of stained cells per square micrometer. The number of stained cells per square micrometer was then averaged across sections for each mouse (*n* = 4–5 per group) and compared across genotypes and conditions by a two-way ANOVA. The number of c-Fos-positive cells was also assessed for correlation between brain regions using a Pearson’s correlation.

### Measurement of intensity

To judge the intensity of the c-Fos staining, we followed procedures previously described by Boushell and colleagues [64]. We used ImageJ to select a rectangular region within the anatomical boundaries described above for each area. Each image was converted to a 16-bit grayscale image. We then measured the mean gray of the selected area as well as the mean gray of the background (area between stained cells). The background mean gray was subtracted from the mean gray of the selected region, and the resulting normalized mean gray was used as an indication of staining intensity. Normalized mean gray was averaged across sections for each mouse (*n* = 4 per group) and compared across genotypes and conditions by a two-way ANOVA.

### RNA sequencing

A separate group of *Fmr1* null and wildtype mice (*n* = 36, 9 per stimulus per genotype) was exposed to the same social stimulus procedure described above for IHC, followed by a 10-h wait period to allow for resulting changes in RNA transcription or processing. Each brain was harvested immediately after rapid decapitation and sectioned in a brain matrix. A 1-mm Palkovits punch was used to collect tissue samples from lateral amygdala and medial amygdala. Samples were obtained from the prefrontal cortex (PFC, including PLC) by razor blade dissection. Samples were then stored at−80 °C prior to total RNA extraction using the Qiagen RNeasy mini kit (Qiagen) according to manufacturer’s protocol. RNA concentration and purity were measured using a NanoDrop ND-1000 spectrophotometer (Thermo Scientific). The Agilent Bioanalyzer 2100 (Agilent Technologies) was used to confirm RNA integrity (RIN greater than 7.0). To reduce variability, tissue samples were pooled across groups with three brain samples per pool and three pools per group. Samples were diluted to result in consistent RNA concentrations across samples within each pool. Following strand-specific library preparation, paired-end sequencing with 30 M reads per library was performed using Illumina HiSeq 2000.

### Differential expression analyses

Reads were mapped to the mouse genome mm10 using TopHat−2.0.10 [65]. The mapping quality for these samples was high, with 89% or more of the reads mapped to the genome. Then, following a standard data analysis protocol [66], we counted the number of reads that fell into annotated genes by samtools−0.1.19 [67] and HTSeq-0.5.4p5 [68] and analyzed the correlation between samples based on normalized counts. The correlation between biological replicates was high for samples from LA and PFC (Additional File 1). For samples from MA, we chose the two replicates with the best correlation from each group. Finally, we performed count-based differential expression analysis using edgeR_3.4.2 [69], which implements general differential analyses based on the negative binomial model. Differential expression analyses were performed for (1) social versus non-social in wildtype, (2) social versus non-social in mutant, (3) mutant versus wildtype in non-social, (4) mutant versus wildtype in social, and (5) the interaction across genotype and condition, which was considered the primary analysis.

### Analyses of differentially expressed genes

Differentially expressed genes (DEGs) from each comparison in each brain region were compared against lists of FMRP target genes [70] and validated autism candidate genes from the Simons Foundation Autism Research Initiative (SFARI) gene database (gene.sfari.org). DEG lists were considered significantly enriched for FMRP or ASD-related genes when a chi-square test revealed overlap at a higher level than chance (*p* < 0.05). Enrichment of differentially expressed genes in specific networks and pathways was discovered using Ingenuity Pathway Analysis (Qiagen), using differentially expressed genes at the *p* < 0.05 alpha level resulting from the comparisons between genotypes and conditions from each brain region. Pathways were considered significantly enriched for differentially expressed genes when *p* < 0.05, with the Benjamini-Hochberg correction for multiple testing applied. Upstream regulators were also assessed using Ingenuity Pathway Analysis, identified by significant enrichment of DEGs in the network of molecules known to be affected by a given regulator (*p* < 0.05).

## Results

### Immunohistochemistry intensity measurements

Immunohistochemistry (IHC) staining intensity analyses for c-Fos revealed significant genotype × stimulus interaction effects for the LA (F(1,12) = 6.48, *p* = 0.026), and MA (F(1,12) = 10.71, *p* = 0.007) but not the PLC (F(1, 12) = 0.044, *p* = 0.838; Fig. 3a–c). Bonferroni post hoc analyses for staining intensity measurements indicated that the social mutant group displayed significantly greater staining intensity than all other groups in both the LA (social mutant group compared to non-social wildtype group, *p* = 0.001; social wildtype group, *p* = 0.002; and non-social mutant group, *p* = 0.006) and the MA (social mutant group copmared to non-social wildtype group, *p* < 0.001; social wildtype group, *p* < 0.001; and non-social mutant group, *p* < 0.001). Main effects for the social stimulus eliciting greater c-Fos intensity were seen across both wildtype and *Fmr1* null mice in the LA (F(1,12) = 12.64, *p* = 0.004) and MA (F(1,12) = 28.94, *p* < 0.001), while main effects for the social stimulus eliciting less c-Fos intensity were seen in the PLC (F(1,12) = 8.11, *p* = 0.015). Genotype main effects were also seen, with overall higher staining intensity in the *Fmr1* null mice compared to wildtype mice in the LA (F(1,12) = 20.97, *p* = 0.001) and MA (F(1,12) = 47.75, *p* < 0.001).

### Immunohistochemistry and cell counts

In contrast to the c-Fos intensity measurements, cell count analyses did not demonstrate interaction effects in any of the brain areas (LA, F(1,12) = 0.085, *p* = 0.776; MA, F(1,12) = 0.03, *p* = 0.868; PLC, F(1,12) = 0.018, *p* = 0.896; Fig. 3d–f). The social stimulus elicited c-Fos expression in a greater number of cells than the non-social stimulus across genotypes in the LA (F(1,12) = 44.05, *p* < 0.001) and the MA (F(1,12) = 8.23, *p* = 0.014). A trend toward a significant effect of social stimulus in the opposite direction was observed in the PLC (F(1,12) = 3.70, *p* = 0.078). Genotype also had a significant effect, with *Fmr1* null mice showing a greater number of c-Fos positive cells in the LA (F(1,12) = 23.73, *p* = 0.001) and the MA (F(1,12) = 18.43, *p* = 0.001), with fewer c-Fos positive cells in the PLC (F(1,12) = 8.97, *p* = 0.011).

Within individual animals, the numbers of c-Fos-positive cells in the LA and MA were highly correlated (*r* = 0.77, df = 10, *p* = 0.003). In contrast, cell counts were strongly negatively correlated between the PLC and LA (*r* = −0.84, df = 10, *p* = 0.0007) and between the PLC and MA (*r* = −0.79, df = 10, *p* = 0.002; Fig. 3k).

### Differentially expressed genes identified by RNA sequencing

**Fig. 4:**
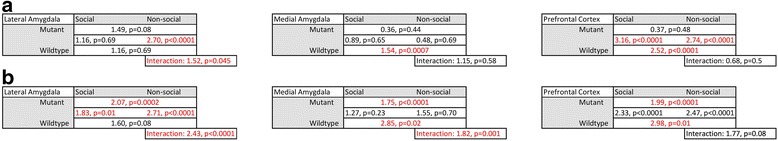
Overlap of differentially expressed genes from *Fmr1* mice with FXS- and ASD-associated genes. Chi-square tests contrasted the list of differentially expressed genes from each comparison (social compared to non-social in mutants, *top line*; social compared to non-social in wildtype, *bottom center*; mutant compared to wildtype in social conditions, *left*; mutant compared to wildtype in non-social conditions, *right*; interaction between genotype and stimulus, *bottom right*) with **a** known FMRP targets and **b** the list of SFARI candidate genes for ASD. Chi-square and *p* values are listed, and contrasts with significant overlap are highlighted in *red text*

RNA integrity was confirmed by RIN assessment (mean ± SEM = 9.07 ± 0.13) prior to RNAseq analysis. Differentially expressed genes (DEGs) from each comparison in each brain region (Additional files 2, 3, and 4) were compared against lists of FMRP target genes [70]. In these comparisons, the *Fmr1* null versus wildtype in the non-social condition would be expected to be the closest representation of gene expression differences driven by genotype alone. This comparison showed a significant enrichment of FMRP target genes among DEGs in the LA (*p* < 0.0001) and the PFC (*p* < 0.0001) but not the MA (*p* = 0.69). Little signal was seen for DEGs enriched for FMRP target genes in the genotype × stimulus interaction analyses, with a nominally significant enrichment (*p* = 0.045) in the LA but not significant enrichment in MA or PFC. In the comparisons of wildtype mice in the social condition and non-social condition, FMRP target genes were enriched above the level of chance in the MA and PFC (*p* < 0.05) but not the LA; whereas these genes were not enriched in the comparisons of mutant mice in the social and non-social condition (Fig. 4a).

DEGs were also compared against validated autism candidate genes from the Simons Foundation Autism Research Initiative (SFARI) gene database (gene.sfari.org). In these analyses, the initial focus was the interaction analyses to identify genes that were differentially affected by the social stimulus in wildtype versus *Fmr1* null mice, which revealed significant enrichment for SFARI genes in the LA (*p* < 0.0001) and MA (*p* = 0.001) and a trend in the PFC (*p* = 0.08). Almost all social versus non-social comparisons within genotype also showed a significant enrichment, suggesting that ASD candidate genes are differentially affected by a social stimulus in these brain regions. Genotype comparisons in both the non-social and social conditions were enriched for SFARI genes in the LA and PFC but not the MA (Fig. 4b).

**Fig. 5:**
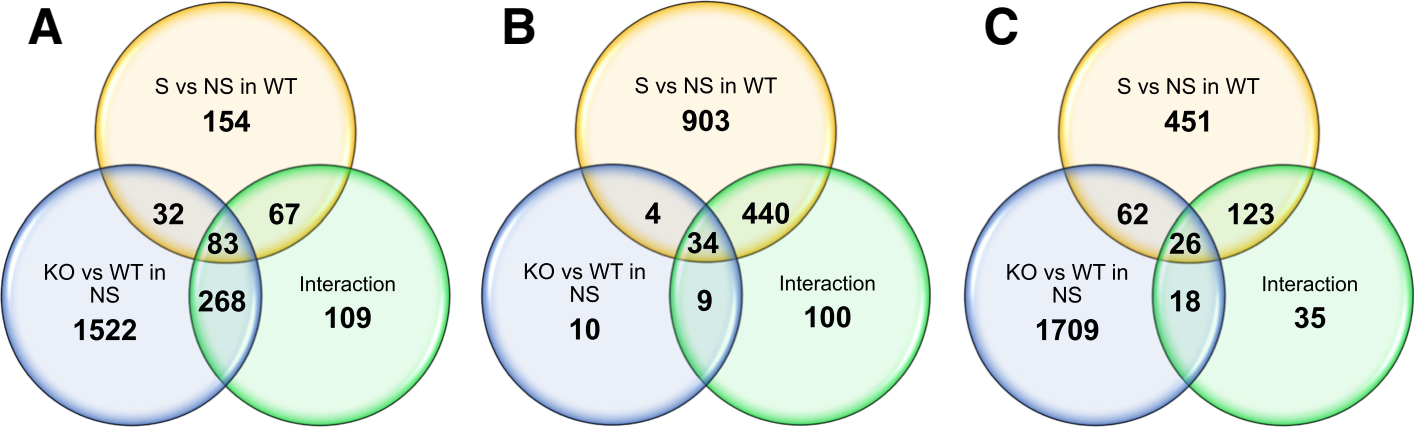
Overlap of differentially expressed genes in baseline comparisons for each brain region. Differentially expressed gene lists (*p* < 0.05) from each baseline comparison (mutants compared to wildtype in non-social conditions in *blue*, social compared to non-social stimulus in wildtype mice in *yellow*) were compared with the list from the interaction analysis of genotype and stimulus variables (*green*) to detect overlap and focus on genes of interest. The number of overlapping genes is shown in corresponding regions of the Venn diagrams for LA (**a**), MA (**b**), and PFC (**c**)

**Fig. 6:**
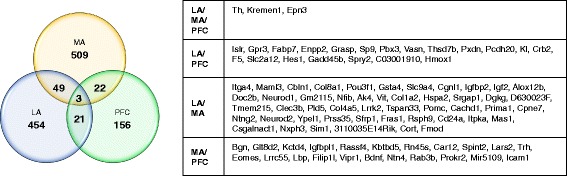
Overlap of differentially expressed genes in LA, MA, and PFC. Differentially expressed gene lists (*p* < 0.05) from the interaction analysis of genotype and stimulus variables were compared from each brain region (LA in *blue*, MA in *yellow*, PFC in *green*). The number of overlapping genes is shown in corresponding regions of the Venn diagram, and the list of overlapping genes for each comparison is shown in the adjoining table

To assess common DEGs across comparisons within each brain region, we evaluated the overlap between gene sets resulting from comparisons of wildtype mice in the social and non-social conditions, the comparisons of mutant and wildtype mice in the non-social condition, and the interaction comparisons (Fig. 5; Additional File 5). In the LA, 83 genes were differentially expressed in all three comparisons (*p* < 0.05). In the MA, 34 genes were differentially expressed in all three comparisons (*p* < 0.05). In the PFC, 26 genes were differentially expressed in all three comparisons (*p* < 0.05). To assess commonalities in DEGs across brain regions, we examined the overlap between interaction comparisons. Three genes, Tyrosine hydroxylase (*Th*), Kringle Containing Transmembrane Protein 1 (*Kremen1*), and Epsin 3 (*Epn3*), were found to be significant DEGs in all three interaction comparisons (*p* < 0.05; Fig. 6). The most common DEGs across comparisons and brain regions are shown in Additional File 6.

### Pathway analyses

A number of canonical pathways exhibited overrepresentation of molecular components within the DEGs (Additional File 7). Within the LA and MA interaction comparisons, enrichment was observed for pathways corresponding to neuronal development (i.e., Axonal guidance signaling *p* = 0.0005 and *p* = 3.24 ×10^−06^, respectively) and intracellular signaling (i.e., AMPK signaling in LA *p* = 0.0032; calcium signaling in MA *p* = 0.0002). Within the PFC interaction comparison, enrichment was seen for inflammatory response (i.e., IL-6 signaling *p* = 0.0035) and intracellular signaling pathways (i.e., TR/RR activation *p* = 0.0009). However, in the LA and PFC, no pathways remained significant when the B-H correction for multiple testing was applied.

**Fig. 7:**
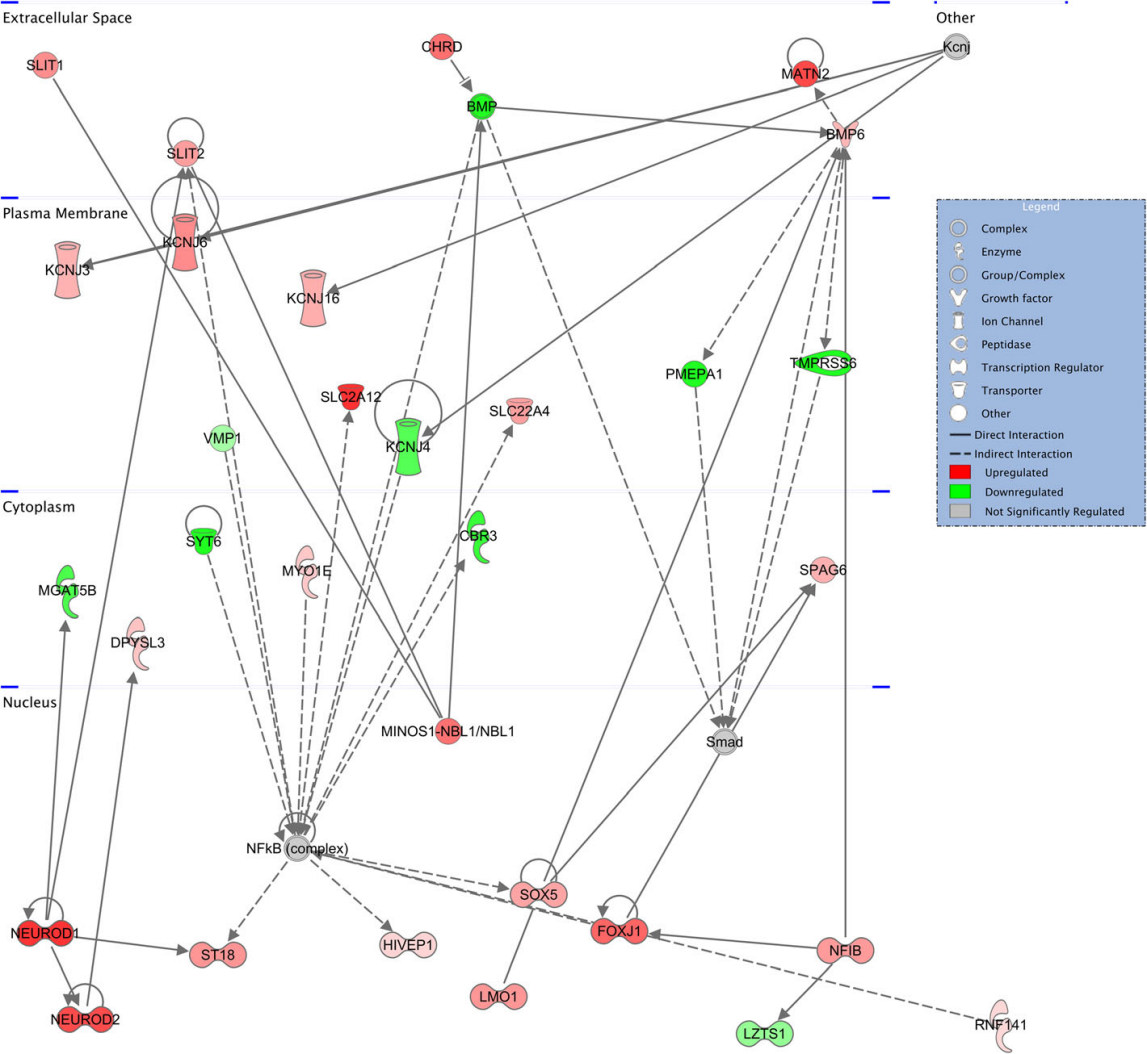
Top network enriched for differentially expressed genes in LA. The network with the greatest enrichment of differentially expressed genes from the interaction comparison of genotypes and stimuli from the lateral amygdala centers on the hub molecule NFkB, which was itself not significantly regulated

**Fig. 8:**
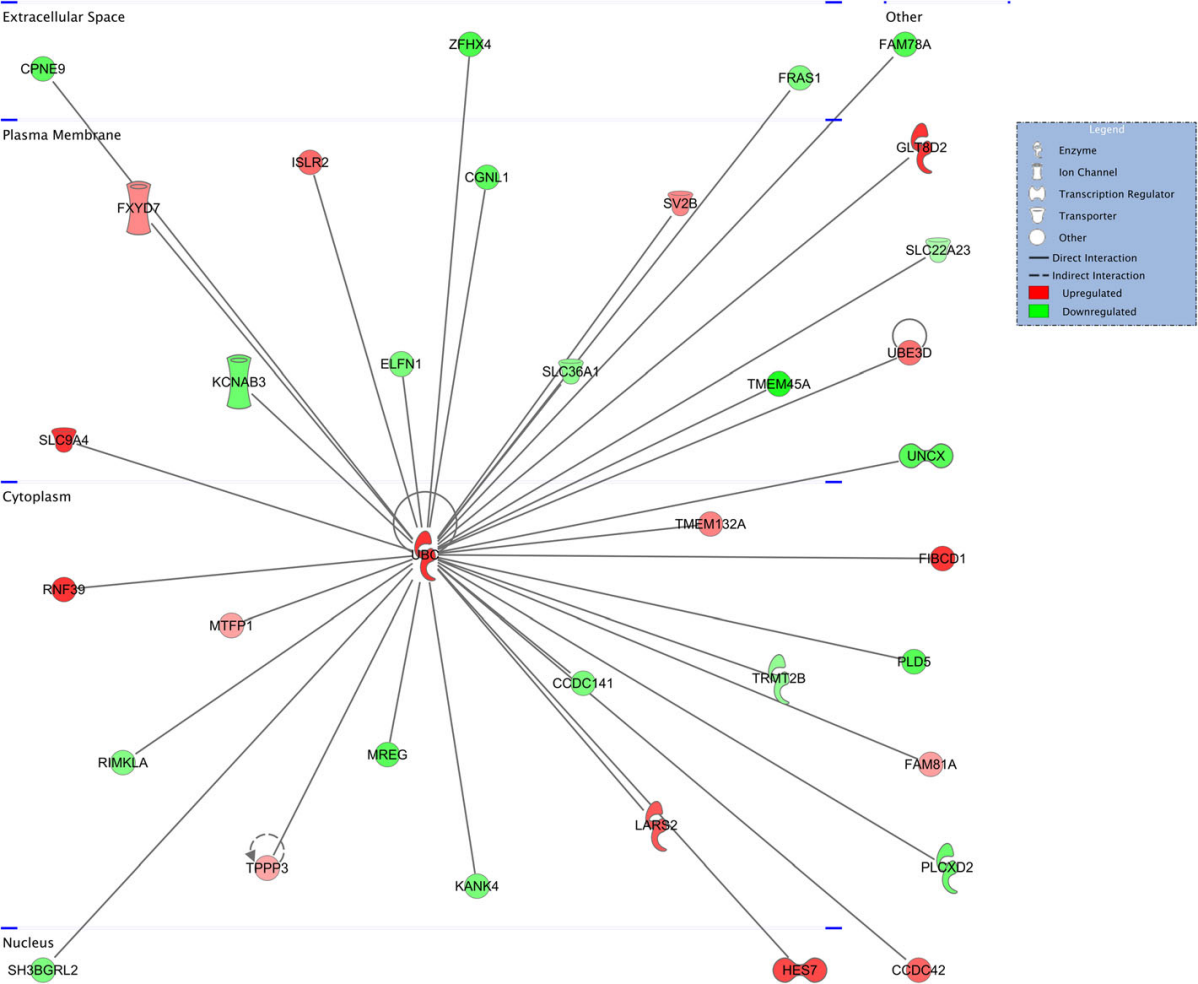
Top network enriched for differentially expressed genes in MA. The network with greatest enrichment of differentially expressed genes from the interaction comparison of genotypes and stimuli from the medial amygdala centers on the hub molecule ubiquitin

**Fig. 9:**
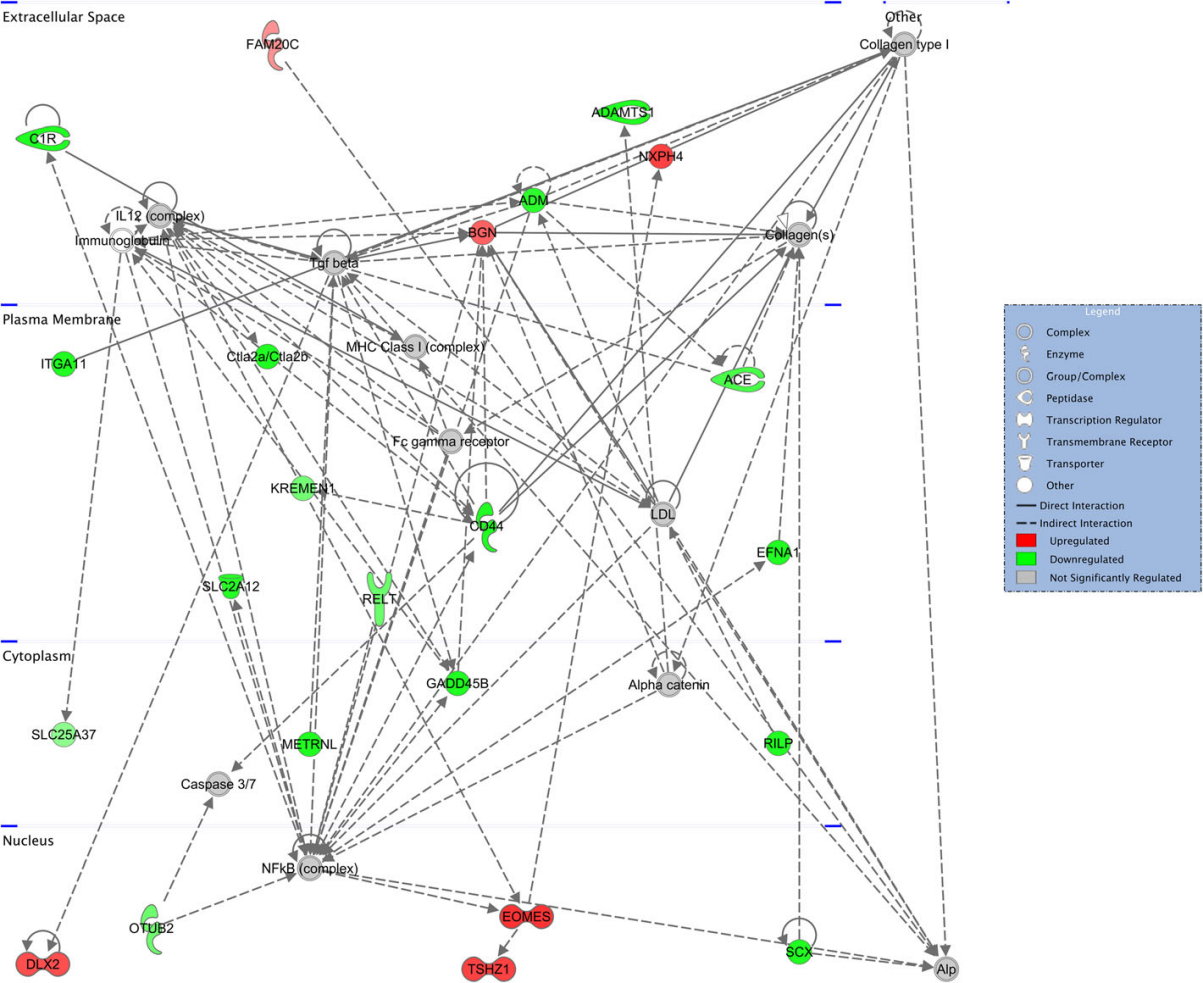
Top network enriched for differentially expressed genes in PFC. The network with the greatest enrichment of differentially expressed genes from the interaction comparison of genotypes and stimuli from the prefrontal cortex centers on the hub molecule NFkB, which was itself not significantly regulated

Network analyses yielded clusters of DEGs whose corresponding proteins are known to interact (Figs. 7, 8, and 9). Enrichment for differentially expressed genes was found in networks associated with nervous system development and function in the LA interaction comparisons, organismal and nervous system development in the MA interaction comparisons, and oxidative stress and developmental disorders in the PFC interaction comparisons. Finally, interaction DEGs were enriched for genes known to be affected by endogenous or exogenous regulators, including beta-estradiol and transforming growth factor beta1, in all three brain regions (3.86 ×10^-17^ ≤ 5.60 ×10^-08^; Additional File 8).

## Discussion

Using the *Fmr1* null mouse as a model of one of the most common ASD-associated syndromes, we were able to identify changes in immediate early gene activation and gene expression following the presentation of a novel mouse, in comparison to a novel object. This may provide insights into pathways and networks underlying altered social response in the *Fmr1* null mice and potentially in people with FXS. Further, the strong overlap with SFARI genes suggests that a social stimulus can be used to provoke changes in gene expression that correspond with ASD-relevant brain regions and molecular pathways within a genetic mouse model of ASD.

Our initial immunohistochemistry in wildtype C57BL/6J mice revealed the LA and MA as having the greatest degree of differential activation, as evidenced by c-Fos levels, in response to a social stimulus compared to a non-social stimulus, while similar findings in *Fmr1* null mice also showed differential activation in the PLC. These findings are in line with recent work by Ferri and colleagues [59], who demonstrated greater levels of immediate early gene products c-Fos and Egr-1 in the BLA of juvenile C57BL/6J mice in response to a social stimulus, as compared with either a non-social stimulus or no stimulus exposure (home cage controls). In contrast, however, they did not observe an increase in c-Fos in response to a social stimulus compared to non-social stimulus in the MA; both the social and non-social stimulus conditions were elevated above the level of home cage controls for the MA. They propose that the MA is more likely to be responsive to various social stimuli after adolescence, which could explain the difference in findings between our studies. In addition, they found only a trend toward a significant difference in c-Fos cell counts in response to stimuli in the PLC, as in our results, though we also found a significant effect of stimulus on staining intensity, and the direction of the effect was the same in both studies, with the social stimulus exposure corresponding to lower c-Fos levels than non-stimulus exposure in the PLC.

In the *Fmr1* null animals, we found hyperactivation of the amygdala of *Fmr1* null animals following the presentation of a novel mouse. These findings suggest that the activation is dysregulated by the deletion of *Fmr1* and support human imaging studies that associate the amygdala with social response in FXS. For example, Watson and colleagues [71] noted increased activation of the left amygdala in response to successive eye gaze exposures in children with FXS compared to controls. Structural imaging suggests a reduction in amygdala volume in FXS [72, 73], and Hoeft and colleagues [74] found that decreased amygdala volume in FXS patients is correlated with altered activation patterns. Recent imaging results in the *Fmr1* null mouse model on the FVB background showed no significant change in the frontal cortex or amygdala volumes, with quite subtle changes overall including primarily white matter, as well as a small increase in ventricle size; although the resolution of MRI for individual brain structures is substantially reduced simply as a result of mouse brain size [75, 76]. The hypoactivation of the PLC in response to the social stimulus was significant for c-Fos intensity but not cell count, but the direction of the effects observed in both measures corresponds with human imaging findings. An fMRI study in children with FXS revealed decreased PFC activation in response to direct eye gaze in comparison to age-matched, typically developing children [71]. Decreased gray matter in the mPFC has also been reported in children with FXS [74].

Our immediate early gene findings also overlap with the broader literature on neural mechanisms of social behavior. For instance, a wide array of data support a role for the medial amygdala in social recognition [77], aggression [78, 79], and mating behavior [80] in mice. The positive correlation between lateral and medial amygdala c-Fos levels in response to a social stimulus align with recent work demonstrating the importance of the intra-amygdalar pathway in processing social behavior and formulating an appropriate response in the context of social fear learning [81]. The high, inverse correlation between amygdala and PLC immediate early gene activation aligns with previous literature suggesting that these two regions communicate during social behavior. The involvement of prefrontal-limbic circuitry, which includes both PFC and the amygdala, has been suggested in several social behaviors in humans including social anxiety [82], social phobia [83], deception [84], social cooperation [85], and empathy [86]. Swartz and colleagues [87] also found a decrease in connectivity between the PFC and amygdala while viewing sad faces as measured by functional magnetic resonance imaging (fMRI). Together with the connectivity and correlational findings between the PLC and amygdala, the findings of lower activation of PLC and higher activation of the amygdala activity in response to a social stimulus suggests dysregulation of at least one node within this circuit in *Fmr1* null mice. This dysregulation in response to a social stimulus may be involved in the differences in social behavior observed in this mouse model of FXS.

The patterns of differentially expressed genes in the LA, MA, and PFC also show convergence with previous data. It is reassuring that this convergence includes highly significant overlap for genes bound by FMRP. Further, it is interesting that this overlap is primarily in the mutant versus wildtype comparison in LA after exposure to the non-social stimulus and in PFC after exposure to either the social or non-social stimulus. The absence of significant overlap with FMRP-bound genes in the genotype comparison in the MA is surprising and could indicate that this is a brain region where FMRP plays less of a direct role in regulating gene expression. Previous work in *Fmr1* null mice has identified changes in electrophysiology and dendritic spine morphology in the basolateral amygdala and the medial prefrontal cortex [88–92]; whereas previous studies have not examined the MA in these animals. These data suggest that the MA could be a brain region that lacks substantial baseline changes due to the direct effects of *Fmr1* deletion but may still show dysregulated activity within a broader circuit, but further work is necessary to examine this possibility. Interestingly, in addition to genotype-driven differences, we also saw a significant enrichment of FMRP-bound genes in the social versus non-social comparison in wildtype animals in both the MA and the PFC, although we did not observe a significant interaction between stimulus and genotype. This suggests that FMRP-bound genes may play an important role in response to a social stimulus, aligning with previous data implicating FMRP as a central hub that regulates genes implicated in ASD [93].

In addition to FMRP-bound genes, which could be expected to show differences in *Fmr1* null mice, we also observed substantial overlap of DEGs with genes implicated in ASD. Our primary analysis for this comparison was the genotype × condition interaction, which showed a significant enrichment for SFARI ASD risk genes for the LA and MA, but only a trend for the PFC. This suggests that a social stimulus may be useful to focus further study on DEGs that are particularly relevant to ASD. Interestingly, *Th*, encoding tyrosine hydroxylase, was one of three genes that was differentially expressed in the genotype × stimulus interaction in all three brain regions. Tyrosine hydroxylase is the rate-limiting enzyme in dopamine synthesis, and the dopamine system has been implicated in FXS, in ASD, and in social response in general. For example, loss of FMRP alters signaling downstream of the dopamine D_1_ receptor [94]. Recent work also shows increased dopamine levels in the striatum of *Fmr1* null mice [27]. De novo and inherited single nucleotide variants have been detected in a number of genes in the dopamine system in ASD. Elegant work using optogenetic and pharmacogenetic approaches has demonstrated that dopaminergic signaling is critical for a number of social functions, including social approach, social reward, and response to social deprivation [95–99]. Beyond the significant interactions, the social versus non-social contrast showed highly significant overlap with the SFARI gene set for *Fmr1* null animals in all three brain regions, further supporting the use of a social stimulus to reveal ASD-relevant DEGs in a genetic mouse model.

Network analysis also pointed toward convergence with previous data, with DEGs clustering in networks that are associated with nervous system development. In addition to further validating the model and social stimulus procedure as producing gene expression differences in pathways relevant for ASD, the analyses may provide focus for future research and potential treatments. By identifying networks in specific brain regions that are differentially regulated in *Fmr1* null mice, we can consider different ways to probe or manipulate the pathway to potentially identify a time during development or adulthood in which perturbation of the dysregulated pathway may rescue specific phenotypes. Additionally, the networks identified may provide specific molecules to consider targeting for treatment, including non-DEGs such as NFκB that occupy a position as molecular hubs interacting with multiple DEGs (Figs. 7 and 9). Finally, the upstream regulator analyses may highlight potential treatments that could reverse the observed pattern of differential gene expression, such as beta-estradiol, which was recently shown to rescue behavioral phenotypes in zebrafish lacking *Cntnap2*, an ASD-risk gene [100]. Most importantly, these pathway and network findings direct us to specific aspects of development, for example axonal guidance signaling or immune response pathways that will be beneficial to examine in *Fmr1* null mice, particularly in relation to their social behavior.

These findings also have some limitations, including the moderately high degree of variability within the RNA sequencing data. Of the three brain areas, biological replicates had a high degree of convergence with the exception of one sample within the medial amygdala that we excluded from all analyses. Additionally, our RNA sequencing approach was focused primarily on pathway and network analyses, with few individual genes reaching significance after correction for multiple testing. We set an *α* level of 0.05 as a threshold for these analyses to balance a desire for specificity with a desire to eliminate false negatives, which could obscure convergence across multiple DEGs. Adjusting the threshold in either direction could yield different findings. Similarly, application of the Benjamini-Hochberg correction at the level of 0.05 for canonical pathway analysis resulted in no enriched pathways in the LA and PFC passing the threshold for significance. These individual DEGs and pathways are therefore exploratory and should be replicated before targeting specific, individual genes.

Another factor to consider is the introduction of the stimulus into the homecage. We minimized the potential for aggression by only housing the test animal in the new cage for 3 days, and containing the stimulus in the pencil cup, but it is possible that the reaction to the stimulus was influenced by territoriality in a way that might be lessened (or increased) by other circumstances.

Finally, the statistical differences between measurements of c-Fos intensity and cell counts should be considered. The effect of the interaction between genotype and stimulus was only significant in c-Fos intensity, although virtually all findings were in the same relative directions between analyses. The significant interaction effect on intensity was due to a larger effect size, and it is possible that this measure is more sensitive to biological variation than cell count. For example, the level of c-Fos expression may vary between cells, leading to cell-specific transcriptional responses. Intensity measurement has the added advantage of being more objective, not requiring an experimenter to apply a threshold as in the cell count. The meaning of each measurement and resulting findings should be considered, but one is not necessarily preferable to the other.

## Conclusions

The findings presented here align with previous research demonstrating the role of the amygdala and the PFC in social behavior in mice and validate the use of the *Fmr1* null mouse for investigating circuitry contributing to social behavior. The use of this well-studied genetic mouse model provides the opportunity to look for overlap with previous findings, potentially serving as a proof of concept for this experimental design focus on the response to a social stimulus. The differential neuronal activation patterns and differential gene expression patterns elicited by a novel social stimulus did intersect with reported neuroimaging findings in fragile X syndrome as well as previous molecular findings in the *Fmr1* null mouse. This approach may therefore prove useful in exploring the neural substrates of social behavior and for identifying molecular pathways contributing to abnormal social behaviors in other mouse models.

## Additional files

Additional file 1: Correlations between biological replicates. The correlations of mapped reads between biological replicates using normalized counts per million for all reads were high for all comparisons in the lateral amygdala (LA) and prefrontal cortex (PFC), but less strong in some replicates in the medial amygdala (MA). MT mutant; WT wildtype; NS non-social; S social. Red *p* < 0.001; white *p* > 0.001. (PDF 144 kb)

Additional file 2: Differentially expressed genes in lateral amygdala (XLSX 5388 kb)

Additional file 3: Differentially expressed genes in medial amygdala (XLSX 5377 kb)

Additional file 4: Differentially expressed genes in the prefrontal cortex (XLSX 5258 kb)

Additional file 5: Overlapping genes of comparisons in each brain region. The differentially expressed genes (DEGs) that overlapped in specific comparisons are listed. The first column indicates DEGs significantly regulated in both the mutant versus wildtype comparison in the non-social condition (MUvsWTinNS) and in the social versus non-social conditions in the wildtype animals (SvsNSinWT). The second column indicates DEGs significantly regulated in both the MUvsWTinNS condition and in the interaction between genotype and stimulus exposure. The third column indicates DEGs significantly regulated in both the SvsNSinWT condition and in the interaction between genotype and stimulus exposure. Overlap in all three comparisons indicates DEGs that were significantly regulated in the MUvsWTinNS, SvsNSinWT, and interaction analyses. Analyses from each brain region are shown on separate sheets, accessed by the tabs at the bottom. (XLSX 37 kb)

Additional file 6: Most commonly differentially expressed genes across all comparisons. The genes that had the most frequent differential expression in analyses of all comparisons in all brain regions are listed, showing where they had significant differences in expression level for a given comparison (X) in each brain region. M mutant; W wildtype; NS non-social; S social. (PDF 270 kb)

Additional file 7: Canonical pathway results for RNAseq data. Canonical pathway analysis revealed pathways with significant enrichment of genes that were differentially expressed in each comparison. The genotype and stimulus condition comparison is shown on the left, with the name of the top 10 enriched pathways, the number of differentially expressed genes that overlap with each pathway, and the adjusted *p* value shown from left to right. Analyses from each brain region are shown on separate sheets, accessed by the tabs at the bottom. (XLSX 18 kb)

Additional file 8: Upstream Regulator results for RNAseq data. Upstream regulator analysis revealed regulatory molecules known to affect a certain network of molecules, overlapping with genes that were differentially expressed in each comparison. The genotype and stimulus condition comparison is shown on the left, with the name of the top 10 upstream regulators, the predicted direction of activation or inhibition based on the up/down-regulation of differentially expressed genes that overlap with the affected network, and the adjusted *p* value shown from left to right. Analyses from each brain region are shown on separate sheets, accessed by the tabs at the bottom. (XLSX 12 kb)

## Abbreviations

ANOVA: Analysis of variance
ASD: Autism spectrum disorder
FMR1: Fragile X mental retardation 1 gene
FMRP: Fragile X mental retardation protein
FXS: Fragile X syndrome
GABA: Gamma-amino butyric acid
ID: Intellectual disability
IHC: Immunohistochemistry
IPA: Ingenuity pathway analysis
LA: Lateral amygdala
MA: Medial amygdala
mGluR: Metabotropic glutamate receptor
PBS: Phosphate-buffered saline
PFC: Prefrontal cortex
PLC: Prelimbic cortex
RNAseq: Ribonucleic acid sequencing
SFARI: Simons Foundation Autism Research Initiative
TBST: Tris-buffered saline with triton
