# Supplementary material for: Effects of a social stimulus on gene expression in a mouse model of fragile X syndrome
Source: Mol Autism. 2017 Jun 23;8:30. doi: 10.1186/s13229-017-0148-6 (PMC5481916; doi:10.1186/s13229-017-0148-6)
Supplement: Supplementary file 1 — Correlations between biological replicates. The correlations of mapped reads between biological replicates using normalized counts per million for all reads were high for all comparisons in the lateral amygdala (LA) and prefrontal cortex (PFC), but less strong in some replicates in the medial amygdala (MA). MT mutant; WT wildtype; NS non-social; S social. Red p < 0.001; white p > 0.001. (PDF 144 kb) [file 13229_2017_148_MOESM1_ESM.pdf]

LA			MA			PFC		
NS.MT.1	0.99	1.00	NS.MT.1	0.92	0.95	NS.MT.1	0.99	0.99
	NS.MT.2	0.99		NS.MT.2	0.98		NS.MT.2	0.99
		NS.MT.3			NS.MT.3			NS.MT.3
NS.WT.1	0.99	0.99	NS.WT.1	0.97	0.99	NS.WT.1	0.99	0.99
	NS.WT.2	0.99		NS.WT.2	0.97		NS.WT.2	0.98
		NS.WT.3			NS.WT.3			NS.WT.3
S.MT.1	0.99	0.99	S.MT.1	0.89	0.94	S.MT.1	0.99	0.99
	S.MT.2	0.98		S.MT.2	0.98		S.MT.2	1.00
		S.MT.3			S.MT.3			S.MT.3
S.WT.1	0.99	0.98	S.WT.1	0.94	0.93	S.WT.1	0.99	0.98
	S.WT.2	0.99		S.WT.2	0.99		S.WT.2	0.99
		S.WT.3			S.WT.3			S.WT.3
