# Supplementary material for: Effects of a social stimulus on gene expression in a mouse model of fragile X syndrome
Source: Mol Autism. 2017 Jun 23;8:30. doi: 10.1186/s13229-017-0148-6 (PMC5481916; doi:10.1186/s13229-017-0148-6)
Supplement: Supplementary file 6 — Most commonly differentially expressed genes across all comparisons. The genes that had the most frequent differential expression in analyses of all comparisons in all brain regions are listed, showing where they had significant differences in expression level for a given comparison (X) in each brain region. M mutant; W wildtype; NS non-social; S social. (PDF 270 kb) [file 13229_2017_148_MOESM6_ESM.pdf]

Most Commonly Differentially Expressed Genes

	Comparisons in Each Brain Area														
	**LA**					**MA**					**PFC**				
Gene Symbol	MvsWinNS	MvsWinS	SvsNSinWT	SvsNSinMU	Interaction	MvsWinNS	MvsWinS	SvsNSinWT	SvsNSinMU	Interaction	MvsWinNS	MvsWinS	SvsNSinWT	SvsNSinMU	Interaction
Epn3	-	X	-	-	X	-	X	-	-	X	-	-	-	-	X
Fabp7	X	-	X	-	X	-	-	-	-	-	X	-	X	-	X
Kremen1	X	-	X	-	X	-	-	X	-	X	-	X	X	-	X
Pbx3	X	-	X	-	X	-	-	-	-	-	X	-	X	-	X
Pomc	X	-	X	-	X	X	X	X	X	X	-	X	-	-	-
Th	-	X	X	X	X	-	-	X	-	X	-	X	X	-	X
